# Plunging Ranula: A Case Report

**DOI:** 10.5402/2011/806928

**Published:** 2010-09-08

**Authors:** Ambika Gupta, F. R. Karjodkar

**Affiliations:** ^1^Department of Oral Medicine, Diagnosis, and Radiology, Government Dental College, Pandit B.D. Sharma University of Health Sciences, Rohtak, Haryana, India; ^2^Department of Oral Medicine, Diagnosis, and Radiology, Nair Hospital Dental College, Mumbai, Maharashtra, India

## Abstract

Plunging ranulas, also known as deep, diving, cervical or deep plunging ranula, usually appear in conjunction with oral ranula. Rarely, these ranulas may arise independent of oral swelling. A rare case of plunging ranula without oral swelling is discussed along with review of literature.

## 1. Introduction

A plunging ranula is an extravasation of saliva from the sublingual gland due to trauma or obstruction of the duct. Fluid from the obstructed gland dissects between the fascial planes and muscle of the base of the tongue to the submandibular space. The exact prevalence of plunging ranula is not known, however, these lesions are considered uncommon. Because most plunging ranulas either accompany a swelling in the floor of mouth or are associated with a history of treatment of intraoral ranula, it is not difficult to diagnose such a lesion. On the other hand, the plunging ranulas in which there is no clinical evidence of an oral connection, needs a diagnostic acumen. The purpose of this paper is to present clinical and radiographic findings of a rare case of plunging ranula without any intraoral extension along with the relevant review of the literature. 

## 2. Case Report

A 50-year-old male reported with a 12-month history of swelling in left submandibular region. The swelling was completely asymptomatic and there was a history of intermittent change in the size of swelling. The patient gave history of surgical intervention and drainage of thick viscous fluid from the swelling in left submandibular region six months back at his native place, by his family physician. However, the swelling reappeared immediately after the procedure. Therefore, the patient visited our institution for definitive management. 

**Figure 1:**
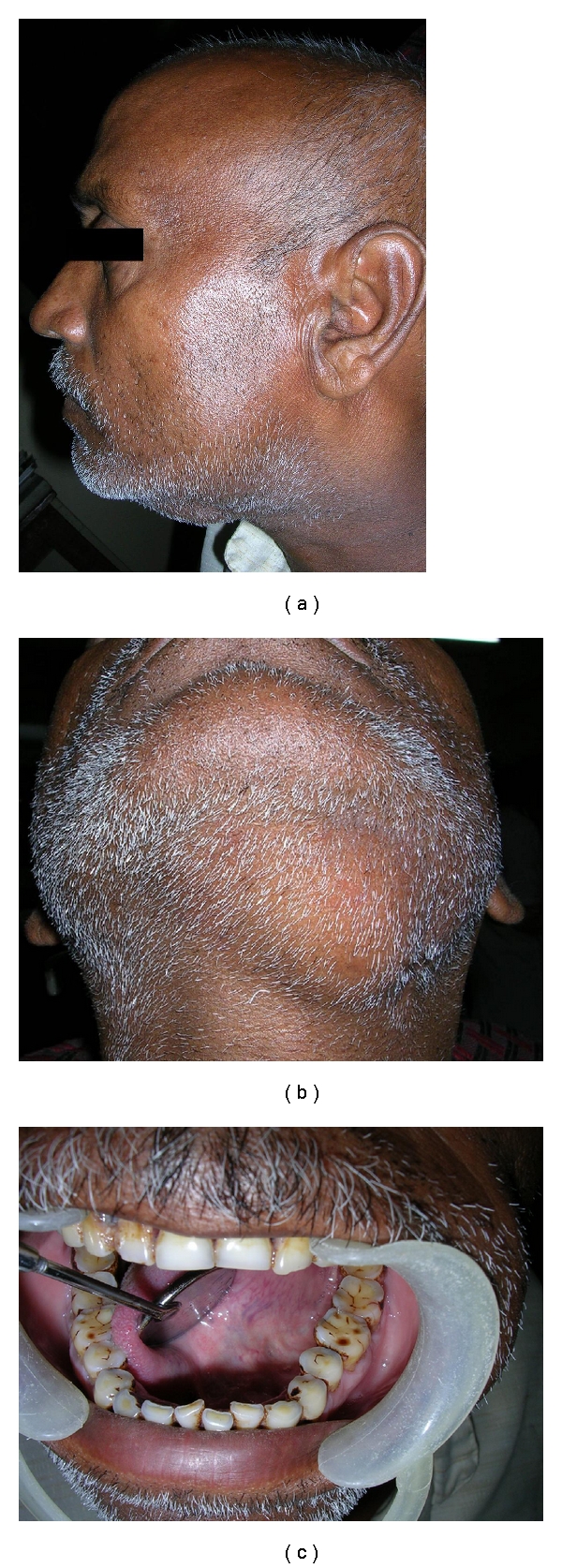
(a) Extraoral profile view showing left submandibular swelling. (b) Extraoral view showing submandibular swelling. (c) Intraoral view showing absence of swelling in floor of the mouth.

The patient was in good health and had no history of any systemic disorder. Family history and personal history were not remarkable. On examination, general condition was good and vital signs were stable. On examination, a diffuse, soft, fluctuant, nontender swelling, about 7 × 5 cm in size, was present in left submandibular region (Figure 1(a)). A scar was visible near left angle of mandible, which was the site of previous surgical intervention (Figure 1(b)). Overlying skin was normal in color and temperature. Intraorally, no swelling was evident in sublingual areas bilaterally (Figure 1(c)). Tooth number 35 was carious. Oral mucosa, gingival, was normal and salivary ducts openings were patent. Oral hygiene was poor.

**Figure 2:**
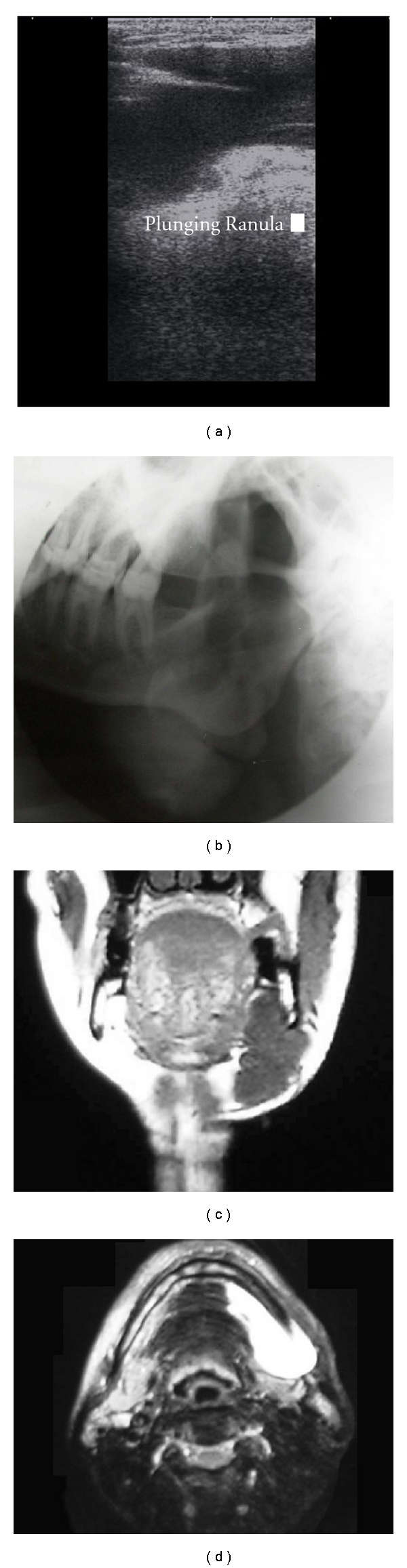
(a) Ultrasonogram showing anechoic fluid collection in left submandibular region. (b) Lateral oblique projection of submandibular region with contrast media injected in sublingual space. (c) Coronal T-1 weighed MR image showing hypointense area of fluid collection above and below the mylohyoid muscle. (d) T-2 weighed axial MRI section showing the extensions of the fluid collection.

Orthopantomograph of the patient revealed generalized horizontal bone loss. Ultrasonography of the swelling showed an anechoic, bilobed fluid collection in left submandibular region with a superficial and deep component (Figure 2(a)). 1.0 ml of a water-based iodine contrast media was injected in left sublingual region and a lateral oblique projection of left submandibular region was made to view the extensions (Figure 2(b)). T-2 weighed MRI images, showed hyperintense fluid filled cavity in left sublingual space, extending to left submandibular space along the posterior edge of mylohyoid muscle (Figures 2(c) and 2(d)).

**Figure 3:**
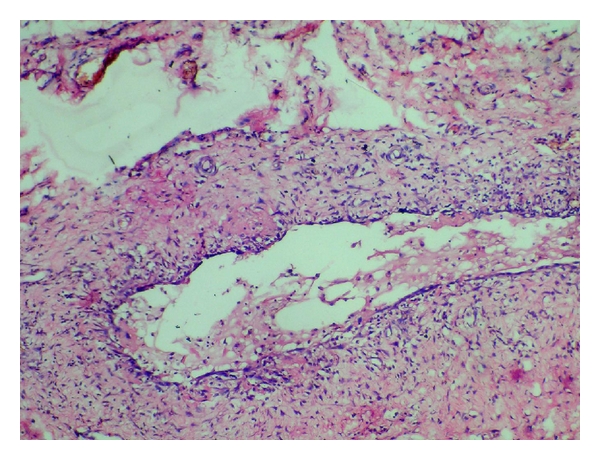
Histopathological picture of the excised lesion showing mucin collection in the lumen lined by connective tissue with inflammatory cells.

Based upon the clinicoradiological and MRI findings, a tentative diagnosis of plunging ranula was made with a differential diagnosis of dermoid and epidermoid cyst, thyroglossal duct cyst, cystic hygroma, and lymphadenopathy. The excision of the lesion was done via cervical approach under general anaesthesia and the tissue was subjected to histopathological evaluation (Figure 3). There was no sign of recurrence for the next six months after which the patient was lost for follow-up.

## 3. Review of Literature

A ranula by definition is a mucus filled cavity, a mucocele, in the floor of the mouth in relation to the sublingual gland [1, 2]. The name “ranula” has been derived from the Latin word “rana” which means “frog.” The swelling resembles a frog's translucent underbelly or air sacs. Ranulas are characteristically large (>2 cm) and appear as a tense fluctuant dome-shaped vesicle, sometimes with a blue hue. The most common site is the lateral floor of the oral cavity. A clinical variant with moderate incidence, plunging ranula occurs when the fluid pressure of the mucin dissects through a perforation in the mylohyoid muscle in the submandibular space [3–5]. Ranulas have a prevalence of about 0.2 cases per 1000 persons and accounts for 6% of all oral sialocysts. The number of ranulas that represents a true retention cyst ranges from less than 1% to 10%. Ranulas usually occur in children and young adults, with the peak frequency in the second decade [6]. The cervical variant tends to occur a little later in the third decade. The diagnosis of a plunging ranula is usually determined by a combination of history, clinical presentation, and imaging studies. 

Plunging ranulas generally appear in conjunction with an oral ranula. Rarely can they arise independently of the oral component. In the absence of oral swelling, the clinical diagnosis of ranula may not be suspected. In upto 45% of the cases, the patient's first presentation is an oral swelling. Plunging ranulas are associated with oral swelling in 34% of cases. Another 21% of the cases occur without any oral involvement. The foremost etiology of ranulas is from the process such as partial obstruction of a sublingual duct. This can lead to formation of an epithelial-lined retention cyst, which occurs in <10% of all ranulas. Second, most common factor is trauma that causes direct damage to the duct or deeper areas of the body of the sublingual gland, leading to extravasation of mucus and formation of pseudocyst. In most cases, it is iatrogenic. Plunging ranulas arise in the neck by one of the following four mechanisms. Firstly, the sublingual gland may project through the mylohyoid, or an ectopic sublingual gland may exist on the cervical side of mylohyoid. This explains most plunging ranulas that exist without an oral component. Secondly, a dehiscence or hiatus in the mylohyoid muscle may occur. This defect is observed along the lateral aspect of the anterior two-thirds of the muscle. Through this defect, the mucin from the sublingual gland may penetrate to the submandibular space. Thirdly, approximately 45% of plunging ranulas occur iatrogenically after surgery to remove oral ranulas. Cases of plunging ranula formation have also been reported secondary to surgical procedures for sialolith removal, duct transposition and implant placement [7–9]. Lastly, a duct from the sublingual gland may join the submandibular gland or its duct, allowing ranulas to form in continuity with the submandibular gland. Therefore, the ranula accesses the neck from behind the mylohyoid muscle [4].

The cervical ranula appears as an asymptomatic, continuously enlarging mass that may fluctuate in size. Most reported ranulas are 4–10 cm in size. The overlying skin is usually intact. The mass is fluctuant, freely movable, and nontender. The mass is not associated with the thyroid gland or lymph node chains. In some instances, detecting salivary gland herniation of a portion of the sublingual gland through the mylohyoid muscle into the neck may be possible. The mass may not be well defined but follows the fascial planes of the neck and may extend into the mediastinum. Similar to the oral ranula, the mass tends to cause a lateral swelling; however, it may cross the midline. They have been reported to extend into the submental region, the contralateral neck, the nasopharynx upto the skull base, the retropharynx and even into the upper mediastinum [10, 11]. Rarely, large-sized ranulas may cause dysphagia or airway obstruction. 

Takimoto suggested a simple radiographic technique for preoperative diagnosis of plunging ranula that involves injecting a contrast media in sublingual space [12]. A sialogram performed on a patient with a sialocyst reveals smooth displacement of the glandular ducts around the mass. No direct communication with the ductal system is demonstrated. However, the best method of demonstrating a communicating cyst is by sialography. 

Ultrasonography is usually inconclusive to study sublingual glands due to their location. On CT, the simple ranula is usually a roughly ovoid-shaped cyst with a homogeneous central attenuation region of 10 to 20 HU. The cystic wall is either very thin or not seen, and the lesion is positioned lateral to the genioglossal muscles and above the mylohyoid muscle. Uncommonly, it can extend anteriorly, behind the symphysis of the mandible, above the genial muscles. The plunging ranula often infiltrates the adjacent tissue planes, extending inferiorly and dorsally to the submandibular gland region, while ventrally it may cross the midline to the contralateral floor of the mouth. Although a plunging ranula may extend into the submandibular triangle and displace the submandibular gland, it does not intrinsically affect this gland [13]. 

MRI is the most sensitive study to evaluate the sublingual gland and its states. On MR imaging, the ranula's characteristic appearance is usually dominated by its high water content. Thus, it has a low T1-weighted, an intermediate proton density, and high T2-weighted signal intensity. This appearance, especially in a plunging ranula, may be similar to that of a lymphangioma, a lateral thyroglossal duct cyst, and possibly an inflamed lymph node. However, if the protein concentration of the ranula's contents is high, the signal intensities can vary, often being high on all imaging sequences. In such cases, the MR differential diagnosis includes entities such as dermoids, epidermoids, and lipomas [14]. 

Histopathologically, the cervical ranula appears identical to the mucus extravasation phenomenon. Biopsy of the lateral part of the neck may reveal only amorphous material with rare inflammatory cells and predominant histiocytes, which stains positive for mucin [2]. Biochemical analysis of fluid shows its high amylase and protein content.

Differential diagnosis of cervical ranula must include thyroglossal duct cyst, branchial cleft cyst, cystic hygroma, submandibular sialadenitis, intramuscular hemangioma, cystic or neoplastic thyroid disease, infectious cervical lymphadenopathy (Epstein-Barr virus, cat scratch disease, tuberculosis), hematoma, lipoma, laryngocele, and dermoid cyst.

Clinicians have been using several different methods for the treatment of cervical ranulas. These include excision of the ranula only, cryosurgery, marsupialization with or without cauterization of the lesion lining, excision of the oral portion of the ranula with the associated sublingual salivary gland or, rarely, the submandibular gland, intraoral excision of the sublingual gland and drainage of the lesion, and excision of the lesion via a cervical approach, sometimes combined with excision of the sublingual gland. Despite these treatments, many patients have experienced recurrence and sometimes larger lesions have occurred. Excision of the ranula with the associated sublingual salivary gland is the most accepted method with low recurrence rate [15]. Risk for paresis and paralysis of the marginal mandibular nerve is the most common complication following surgical therapy of ranula [16]. A biopsy of the cystic wall is recommended not only for histologic confirmation, but also to rule out presence of squamous cell carcinoma arising from the cyst wall and papillary cystadenocarcinoma of the sublingual gland, which may present as ranula.

Besides surgical management, CO_2_ laser has been used to vaporize ranulas [17]. In rare cases, radiation therapy is an alternative. Low doses of 20–25 gray are effective. Intracystic injection of the streptococcal preparation, OK-432, has been used to treat this lesion in a few reported cases. The use of this sclerosing agent as a treatment approach for the cervical ranula is considered experimental [18, 19]. A recent study found orally administered Nickel Gluconate-Mercurius Heel-Potentised Swine Organ Preparations D10/D30/D200, a homotoxicological agent to be an effective treatment modality for ranulas [20].

## 4. Discussion

In our case, the patient presented with a swelling that was restricted to left submandibular region and the patient was completely asymptomatic. On injecting a contrast medium in the sublingual space, we could appreciate the extension of the dye in left submandibular space, which was suggestive of a plunging ranula. Although, Ultrasonography is usually nondiagnostic, the USG for this patient showed a bilobed fluid collection in left submandibular region with a superficial and deep component. MRI, which is the most sensitive imaging modality for studying ranula, showed hyper intense fluid filled cavity in left sublingual space, extending to left submandibular space along the posterior edge of mylohyoid on T-2 weighed images. T-1 weighed images showed a well-defined hypointense area suggestive of plunging ranula (Figure 2). The patient was referred to Department of Oral and Maxillofacial Surgery for surgical management, where the lesion was excised via cervical approach under general anaesthesia. The excised tissue was sent for histopathological evaluation. Histopathology revealed a connective tissue with inflammatory cell response, lining the lumen. The lumen showed areas of spilled mucin containing histiocytes (Figure 3). The overall findings were consistent with the diagnosis of plunging ranula. 

## 5. Conclusion

Though the cases of plunging ranula have been documented with moderate frequency, failure to differentiate the clinical features of oral and plunging ranulas may be a diagnostic pitfall. These lesions may be difficult to differentiate from benign and malignant salivary gland tumors, especially cystadenocarcinoma and mucoepidermoid carcinoma. A case of squamous cell carcinoma in the wall of ranula has also been reported [21]. So, thorough radiological, biochemical, and histopathological investigations should be carried out for all cases of suspected plunging ranulas.
